# Simulate_PCR for amplicon prediction and annotation from multiplex, degenerate primers and probes

**DOI:** 10.1186/1471-2105-15-237

**Published:** 2014-07-09

**Authors:** Shea N Gardner, Tom Slezak

**Affiliations:** 1 Computations/Global Security, Lawrence Livermore National Laboratory (LLNL), Livermore, CA 94550, USA

**Keywords:** PCR target prediction software, HIV 1, Coronaviridae, Multiplex PCR, Amplicon prediction, Degenerate PCR, Consensus PCR

## Abstract

**Background:**

Pairing up primers to amplify desired targets and avoid undesired cross reactions can be a combinatorial challenge. Effective prediction of specificity and inclusivity from multiplexed primers and TaqMan®/Luminex® probes is a critical step in PCR design.

**Results:**

Code is described to identify all primer and probe combinations from a list of unpaired, unordered candidates that should produce a product. It predicts and extracts all amplicon sequences in a large sequence database from a list of primers and probes, allowing degenerate bases and user-specified levels of primer-target mismatch tolerance. Amplicons hit by TaqMan®/Luminex® probes are indicated, and products may be annotated with gene information from NCBI. Fragment length distributions are calculated to predict electrophoretic gel banding patterns.

**Conclusions:**

*Simulate_PCR* is the only freely available software that can be run from the command line for high throughput applications which can calculate all products from large lists of primers and probes compared to a large sequence database such as nt. It requires no prior knowledge of how primers should be paired. Degenerate bases are allowed and entire amplicon sequences are extracted and annotated with gene information. Examples are provided for sets of TaqMan®/Luminex® PCR signatures predicted to amplify all HIV-1 genomes, all *Coronaviridae* genomes, and a group of antibiotic resistance genes. The software is a command line perl script freely available as open source.

## Background

PCR-based methods of detection and amplification require that primers be target-specific. PCR is used in single- or multiplexed molecular diagnostics [1,2], consensus PCR, and target enrichment prior to sequencing [3] with, for example, Ion AmpliSeq™ (Life Technologies) [4], TruSeq Amplicon (Illumina), and Haloplex (Agilent) [5]. To predict desired reactions with target and undesired cross reactions, simulate_PCR software is described to calculate the possible primer pairings and products of PCR (primer only) or primer + probe triplet reactions (as in TaqMan® or Luminex®) in either single-plex or complex multiplex reactions, as compared to a large sequence database. We use it daily for applications in molecular diagnostic signature development and erosion analysis (i.e. checking robustness of PCR-based signatures in the face of new sequence data), and have used it in the process of developing primer panels with more than 1000 primers for target enrichment prior to sequencing.

*Simulate_PCR* is the only software that 1) finds all possible primer pairings predicted to result in a product from an unordered, unpaired list of primer and probe candidates, 2) can be run from the linux command line for high throughput applications, 3) can calculate products from multiplexed combinations of many primers, 4) predict probes binding to those amplicons, 5) allows degenerate bases, 6) extracts entire amplicon sequences and creates a FASTA file of amplicons for downstream analyses, and 7) automatically annotates amplicons with gene information downloaded from NCBI’s Genbank for those matches with a gi number.

Other tools to simulate PCR exist, like NCBI *Primer-BLAST*[6], *FastPCR*[7], and *csPCR*[8]. They are excellent for low throughput analyses where manual inspection and a graphical user interface (GUI) is desired, and they offer a number of very useful features not offered by *simulate_PCR*, such as primer design, and primer T_m_, secondary structure and primer-dimer prediction. However, when the goal is prediction of target and off-target amplification and probe binding as part of an automated pipeline, with optional amplicon sequence extraction and gene annotation, *simulate_PCR* provides a unique capability. For example, if a long list of primer candidates has been designed with desired thermodynamic or taxonomically specific characteristics, it can be a chore to identify the best pairings if one has to check all possible pairwise combinations using a tool that considers only one single-plex primer pair at a time to predict products, as with the web tool *Primer-BLAST*. Another tool, *FastPCR* claims multiplex capabilities, but unfortunately this capability only applies to primer design (e.g. avoiding primer dimers) but not to amplicon prediction from mixtures of more than 2 primers in the same reaction. Moreover, it is not freely available except as a trial version for Windows and it must be run from a GUI, making it unfeasible for large scale unix/linux applications. The *csPCR* program does predict amplicons from multiplexed primers, but it must be run from a GUI, suitable for one-off analyses by non-programmers, but presenting a problem for high throughput automated pipelines. *MFEprimer2.0* has a command line version and can take a batch of primers with unspecified pairings, but requires a large amount of memory so that most users will not have sufficient RAM to compare to the NCBI nt database. When we tried several examples on the *MFEprimer2.0* website, which *simulate_PCR* ran in a few seconds, they never finished after several hours, although this could also have been a web server issue on the days we tried. In summary, none of the currently available PCR target prediction tools have the capability to consider primer + probe triplet reactions, none extract entire amplicon sequences, instead reporting only the positions of primer matches, none annotate gene information that overlaps the amplicons, none handle large numbers of unpaired, multiplex, degenerate primers compared to large sequence databases, and most cannot be run as part of an automated pipeline without a GUI or web interface. Thus, *simulate_PCR* addresses an unmet need to aid in the design and assessment of PCR-based molecular diagnostic signatures.

Simulate_PCR is an open source, command line Perl script that calls the *BLAST*[9] programs *makeblastdb*, *blastn*, and *blastdbcmd*, and the NCBI *efetch* utility (http://www.ncbi.nlm.nih.gov/books/NBK25498/#chapter3.ESearch__ESummaryEFetch). It can be run after multiplex degenerate primer and probe design using software such as *PriMux*[10], which automatically creates a FASTA file of primers and probes formatted as input for simulate_PCR.

## Implementation

### Approach

The script *simulate_PCR* predicts PCR amplicons from a sequence database given primers and, optionally, probes. Multiplex degenerate primers encoded with IUPAC characters are *BLAST*ed against sequences in the database, and amplicons calculated from primer-database sequence matches with no more than the allowable number of mismatches that are in the correct orientation and distance. If the input file contains probes, indicated with “|IO” (Internal Oligo) at the end of the FASTA defline, probe matches are reported within the amplicons. If the option -extract_amp is set to 1, then amplicon sequences are extracted with *blastdbcmd*.

Any non-matching BLAST position is a mismatch, including SNPs, indels, and degenerate bases. These break up *blastn* search seeds, so more sensitive detection can result from including all primer/probe variants instead of degenerates. Another way to trade sensitivity for speed is by setting the maximum number of blast hits to report per primer with the option -max_target_seqs. This should be as least twice as high as the expected number of sequences targeted, and can be much higher if signature components are highly conserved. The third way to alter sensitivity is to modify the –word_size option. Default is 4, the minimum allowable *BLAST* seed size, so this value can only be increased for faster speed.

**Figure 1:**
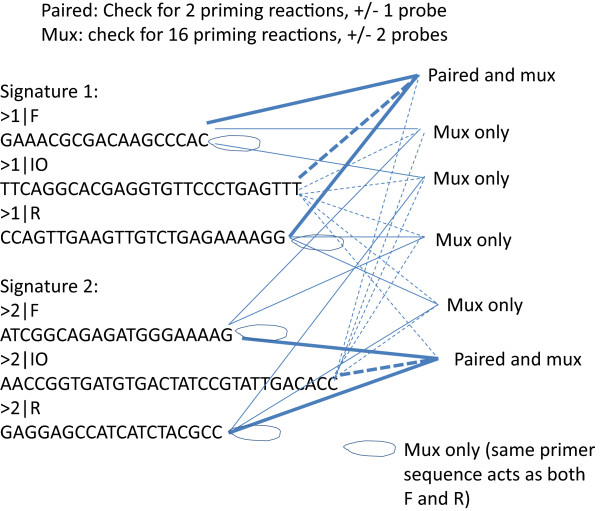
**Diagram of the possible primer pairings allowed in the paired versus mux predictions of *simulate_PCR.*** Heavier lines indicate F/R/IO combinations considered for paired reactions, and all lines indicate pairings considered for mux, which allows any combination of primers to pair up in a reaction with either primer behaving as the forward or reverse. Dashed lines indicate checks for a probe to land on an amplicon produced by a given primer pair. In the paired setting, primers listed as “|F” must act as forward primers and as “|R” as reverse primers, while in the mux setting either may act as forward and/or reverse. From a given set of *BLAST* results, the paired setting checks for two possible priming reactions of signature 1 and signature 2, and reports hits both with and without the probe. Mux checks for 16 possible priming reactions and reports all hits to either probe.

By setting the *simulate_PCR* –mux option to 0 the code predicts specific forward-reverse primer pairings as a singleplex PCR or primer/probe triplet reaction. Setting –mux 1 predicts amplicons from any combination of primers and probes, as illustrated in Figure 1.

With the option –genes 1, if any sequences have gi numbers, annotations which overlap predicted amplicons are automatically downloaded from NCBI Genbank with the *efetch* utility. Annotations reported are primary tag, product, gene, note, and protein id, for coding sequences (CDS), genes, mRNA, mature peptides, miscellaneous features, etc.

Inputs are a file of primers and probes and a file of sequences (either FASTA or *BLAST* database) to be checked for amplicons. Additional command line parameters are minimum and maximum reportable amplicon lengths, the maximum number of mismatches allowed per primer or probe compared to a database hit, whether to report gene annotations, whether to run as single- or multiplex, whether to extract amplicon sequences, the number of blastn threads, the maximum number of *BLAST* hits to report per query (*blastn* option -max_target_seqs), the *blastn* word size (−word_size) and *blastn* evalue.

### Output files

A number of output files are produced: An “amplicons” file of tab-delimited text contains predicted targets hit, amplicon sequence, length, position, primer and probe combination, number of degenerate bases and mismatches per primer/probe, probe position on amplicon, probe on plus or minus strand, and gene annotations overlapping the amplicon, with multiple annotations separated by three colons (examples at https://sourceforge.net/projects/simulatepcr/files/). The “amplicon_distribution” file summarizes fragment length distributions for each target, useful to predict electrophoretic gel banding patterns. A FASTA file contains the predicted amplicons, which will be empty if –extract_amp option is 0. The “detection_counts_by_triplet.#mismatches” files list all forward/reverse primer pairs and probe triplets with the number of sequences in the database each is predicted to detect. These detection counts are calculated assuming 0 mismatches between primers/probes and the database sequence, or up to 1 mismatch per primer or probe, etc. up to the maximum number of mismatches specified in the input –mm option. Results from each mismatch count are shown in separate files. The file “one_primer_but_no_amp” lists the database sequences for which one primer matched without a paired primer within the allowable amplicon length. There is a README and an example at https://sourceforge.net/projects/simulatepcr/files/.

### Computational examples

Primer and probes were predicted for 3 target sets: 1) 547 *Coronaviridae* genomes; 2) 1809 HIV-1 genomes; 3) 9 antibiotic resistance gene sequences (Table 1). 1) and 2) were the set of all available genomes downloaded from Genbank on August 5, 2013, and 3) was several of the first sequences listed in a download of the genes from the Comprehensive Antibiotic Resistance database [11] plus an ndm-1 antibiotic resistance gene sequence from NCBI. Primers and probes were designed using the *run_Primux_triplet* script from *PriMux* (https://sourceforge.net/projects/primux), with the options in Additional file 1. Primers and probes for HIV are provided in Additional file 2, for *Coronaviridae* in Additional file 3, and for antibiotic resistance genes in Additional file 4. *Simulate_PCR* was run with options –minlen 30 –maxlen 1000 –mm 3 –mux 1 –num_threads 12 -word_size 4 -evalue 1000 , and –max_target_seqs 1000 for *Coronaviridae* and antibiotic resistance genes or –max_target_seqs 10,000 for HIV. Primers were compared against original genomes to assess target inclusivity and against the NCBI nt database (from December 2013) for off-target reactions.

**Table 1:** Antibiotic resistance target genes

**Source organism**	**Gene**
*Klebsiella pneumoniae*	Sul1
*Bacteroides fragilis*	tetX
*Azoarcus sp. BH72*	czcA2
*Mycobacterium tuberculosis CDC1551*	MT3777
*Azoarcus sp. BH72*	oprM3
*Klebsiella pneumoniae*	blaSHV-167
*Klebsiella pneumoniae*	SHV-133
*Klebsiella pneumoniae*	blaSHV-72
*Klebsiella pneumoniae*	NDM1

## Results and discussion

Results are summarized in Table 2 and available from https://sourceforge.net/projects/simulatepcr/files/. For HIV1, the 14 degenerate primers are predicted to amplify all targets in the original FASTA file with various combinations of forward and reverse primer pairings. *Simulate_PCR* predicted over 12,000 unique sequences amplified from nt from the combined set of primers and probes. All of the database sequences amplified are HIV except some synthetic constructs and vectors and 3 SIV sequences: gi|402243571 isolate LB715, gi|387119180 clone SIVcpzMB897.c2, and gi|387119170 clone SIVcpzEK505.c2. Each of these SIV sequences had mismatches to the probe, so should not be detected by a TaqMan reaction with stringent conditions that exclude hybridization with mismatches. Two of the SIV sequences had human-specific adaptations in gag [12], so these signatures may be specific for HIV *or* human adapted SIV. The ranked list of triplets from the nt database shows that the combination 1|F, 2|R, 78|IO is predicted to detect 5939 unique database sequences with 0 mismatches between the triplet components and the sequence. In total, there are 258,604 predicted PCR products.

**Table 2:** **Summary of timing, memory, and numbers of predicted hits for *Coronaviridae *** **and HIV1 signatures**

**Target set**	**No. of target seqs.**	**No. of primers**	**No. of probes**	Time against input fasta database* (h:mm:ss or m:ss)	**Time against nt**	**RAM used (MB) against input fasta**	**RAM used (GB) against nt**	**Number of targets amplified**	**Number of targets with probe on amplicon**	**Number of sequences amplified from nt**	**Number of sequences amplified from nt outside the target group**
*Coronaviridae*	547	64	45	1:29	3:36:39	340	10.9	547	541	1091	2 vectors
											1 Glaucous-winged gull^+^
HIV1	1809	14	96	4:25	4:10:44	881	14.5	1809	1809	12371	8 synthetic contructs
											10 vectors
											3 SIV

*Timings using 12 CPU on Intel Xeon 5660 processor. Timings were with options –genes 0 -extract_amp 0.

^+^Hit to Glaucous-winged gull had 1 mismatch in forward primer and no matching probes.

There were ~39,000 sequences with one primer match but no paired primer within the reportable length range. A single 18-24-mer with up to 3 mismatches is likely to match many sequences. The PCR specificity stems from the requirement for 2 matches within priming distance and orientation. However, one cannot always count on primer pairing specificity, making it essential to check for unanticipated primer pairing combinations in a multiplex that could amplify undesired non-target sequences.

For *Coronaviridae* primers, one amplicon from a glaucous-winged gull was predicted if a single mismatch in the forward primer was allowed, although there were no matching probes. This was the only predicted amplicon from nt outside *Coronaviridae*, besides synthetic RNA transcription vectors derived from coronaviruses (feline coronavirus and murine hepatitis virus vectors pBRDI1 and pBRDI2). The run took just over a minute against the *Coronaviridae* genomes database, 4.5 hrs or 3.5 hrs against nt with or without amplicon extraction and gene annotation, respectively.

There were 14 primers and 6 probes for the 9 antibiotic resistance genes. *Simulate_PCR* compared to the original target sequences finished in 8 seconds. Comparing to nt required 47 minutes or 55 minutes, excluding or including the steps to extract amplicons and annotate genes, respectively. Predictions indicate that the primers and probes should amplify 1573 sequences (Additional file 1: Table S1), including (in addition to those organisms in Table 1), *Achromobacter denitrificans*, *Enterobacter cloacae*, *Mycobacterium bovis*, *Acinetobacter baumannii*, *Citrobacter freundii*, *Enterococcus faecalis*, *Shigella dysenteriae*, *Yersinia pestis*, *Pseudomonas aeruginosa*, various vectors, plasmids, and uncultured bacteria, and many others.

## Conclusions

A simple script called *simulate_PCR* is described to identify combinations of primer pairs and primer/probe triplets that detect database sequences, rank the pairs and triplets by the number of sequences they detect, extract amplicon sequences and gene annotations, and predict fragment length distributions from the multiplex. Degenerate bases are allowed, as are a user-specified number of mismatches between triplet components and database sequences. The tool allows a user to specify desired primer pairings, or with the “mux” setting it checks all combinations of primers and probes. This enables a user to input an unordered, unpaired list of candidate forward and reverse primers and probes and let the results guide which pairings achieve the desired specificity. Simulate_PCR is a command line script, so can be run as part of a pipeline where a GUI is not suitable. Our group has used this tool to predict products from multiplexed sets with thousands of primers compared against large sequence databases of all available whole finished and draft microbial genomes (75 GB) and against nt. Extracting a FASTA file of the amplicon sequences is convenient when analyzing high throughput sequencing data that has undergone target enrichment. For example, running *simulate_PCR* on an AmpliSeq™ primer pool with thousands of primers provides an ideal FASTA database for read mapping the resulting sequence data.

Some changes to alter the sensitivity/speed tradeoff of *simulate_PCR* could include swapping out *blastn* with a short read mapper to improve speed, or with more sensitive alignment such as FASTA [13] or Smith-Waterman, at the cost of slower speed. Using a k-mer index algorithm to find primer binding sites could improve speed and sensitivity, but requires large amounts of RAM for large sequence databases. This is the technique used by *MFEprimer*, which requires 12 GB RAM for a 3 GB human genome database [14], implying that larger databases like nt or a 75 GB microbial genome database would not be feasible with the RAM available to most users. However, we have tuned the default blastn parameters in *simulate_PCR* to maximize sensitivity, and found that with the threaded option it performs very fast for situations with up to several thousand primers and probes. Command line options can be adjusted to balance tradeoffs of sensitivity versus speed. *BLAST* heuristics may fail to report all matches to short oligos if degenerate bases break matches below the word length of 4, which is only a problem with large numbers of degenerate or mismatching bases. In this case, one could replace degenerate primers with the expanded variant combinations to improve sensitivity at the expense of speed.

The optional steps of amplicon extraction (*blastdbcmd*) and gene annotation of amplicons (*efetch*) can be time-intensive, and it is recommended that they be omitted if one expects results with more than 10,000 amplicons. The advantage of *blastdbcmd* is that it allows one to extract amplicon sequences from a precompiled blast database (e.g. nt) without the extremely large FASTA input file from which the *BLAST* database was created. The original FASTA data may not even be available if only the precompiled nt blast database was downloaded from NCBI.

In summary, a command line Perl script was developed to predict products from single- or multiplex degenerate PCR or TaqMan®/Luminex® reactions compared against large sequence databases. *Simulate_PCR* is useful to select primer pairings and probe combinations, check for desired target amplification and non-target cross-reactions against databases tens of GB in size, rank pairs and triplets by the number of sequences they are predicted to detect, predict fragment length distributions from a multiplex, extract amplicon sequences, and annotate genes overlapping them. We use this code daily to predict the specificity and target inclusivity of molecular diagnostic signatures and multiplexes for target enrichment prior to sequencing.

## Availability and requirements

**Project name:** s*imulate_PCR*

**Project home page:**http://sourceforge.net/projects/simulatepcr

**Operating system(s):** Platform independent

**Programming language:** PERL

**Other requirements:** Installed and in path: *blastdb*, *blastn*, *blastdbcmd*, and NCBI *efetch* utility if amplicon annotation is desired

**License:** BSD

**Any restrictions to use by non-academics:** none

## Competing interests

The authors declare that they have no competing interests.

## Authors’ contributions

SNG wrote the code and SNG and TS wrote the manuscript. Both authors read and approved the final manuscript.

## Supplementary Material

Additional file 1 Primer and probe design parameters, and Table S1 listing species or sequence elements from nt that the antibiotic resistance primers are predicted to detect. Click here for file

Additional file 2 Primer and probe sequences for HIV-1. Click here for file

Additional file 3 **Primer and probe sequences for *Coronaviridae.***
Click here for file

Additional file 4 Primer and probe sequences for selected antibiotic resistance genes. Click here for file
