# Supplementary material for: Simulate_PCR for amplicon prediction and annotation from multiplex, degenerate primers and probes
Source: BMC Bioinformatics. 2014 Jul 9;15:237. doi: 10.1186/1471-2105-15-237 (PMC4226945; doi:10.1186/1471-2105-15-237)
Supplement: Additional file 1 — Primer and probe design parameters, and Table S1 listing species or sequence elements from nt that the antibiotic resistance primers are predicted to detect. [file 1471-2105-15-237-S1.docx]

**Supplementary information**

Primer and probe design methods

Primux was set to generate conserved but not genotyping probes. Up to 3 degenerate bases per primer or probe were allowed. Thus, the script attempted to design a minimal set of conserved, degenerate primers such that at least one amplicon with an internal probe could be generated from every genome in the target set.

**PriMux options file for primer selection:**

-verbose=0

-dir=results

-write_logfile=1

-write_kmer_counts=0

-write_kmer_locations=0

-primer_selection_iterations=1

-input_sequences=fasta

-max_mm=3

-topN_min=50

-topN=1

-bottomN=10

-count_all=0

-topN_pp=100

-topN_ss=100

-min_kmer_len=18

-max_kmer_len=24

-kmer_len_inc=1

-min_amplicon_length=80

-max_amplicon_length=250

-permit_wobbles=0

-maxPolyX=4

-use_fast_tm_calc=0

-use_gc=1

-use_entropy=1

-use_unafold=1

-min_entropy=3.5

-Na_conc=0.2

-Mg_conc=0.0015

-anneal_temp=37.0

-strand_A_conc=2.5e-08

-strand_B_conc=2.5e-08

-min_hairpin_dG=_5.0

-min_primer_dimer_dG=_6.0

-min_tm=60.0

-max_tm=65.0

-min_percent_gc=20.0

-max_percent_gc=80.0

-min_dist_mm_to_3prime_end=3

-run_find_amplicons=1

**PriMux options file for probe selection**

-verbose=0

-dir=results_probes_conserved

-write_logfile=1

-write_kmer_counts=0

-write_kmer_locations=0

-primer_selection_iterations=1

-probe_selection_iterations=1

-input_sequences=amplcn

-max_mm=3

-topN_min=50

-topN=1

-bottomN=50

-count_all=0

-topN_pp=100

-topN_ss=100

-min_kmer_len=18

-max_kmer_len=30

-kmer_len_inc=1

-min_amplicon_length=80

-max_amplicon_length=250

-maxPolyX=4

-permit_wobbles=0

-use_fast_tm_calc=0

-use_gc=1

-use_entropy=1

-use_unafold=1

-min_entropy=3.5

-Na_conc=0.2

-Mg_conc=0.0015

-anneal_temp=37.0

-strand_A_conc=2.5e-08

-strand_B_conc=2.5e-08

-min_hairpin_dG=_5.0

-min_primer_dimer_dG=_6.0

-min_tm=68.0

-max_tm=73.0

-min_percent_gc=20.0

-max_percent_gc=80.0

-min_dist_mm_to_3prime_end=0

**Table S1:** Species or constructs from nt that are predicted to be detected by the antibiotic resistance primers, allowing up to 3 mismatches between primer and target.

Achromobacter_denitrificans

Achromobacter_xylosoxidans

Acinetobacter_baumannii

Acinetobacter_baylyi

Acinetobacter_bereziniae

Acinetobacter_calcoaceticus

Acinetobacter_haemolyticus

Acinetobacter_johnsonii

Acinetobacter_junii

Acinetobacter_lwoffii

Acinetobacter_pittii

Acinetobacter_sp._NFM2

Acinetobacter_sp._SUN-72

Aeromonas_caviae

Aeromonas_hydrophila

Aeromonas_salmonicida

Aeromonas_sp._GK7

Aeromonas_sp._P2G1

Arabidopsis_thaliana

Arthrobacter_arilaitensis

Azoarcus_sp._BH72

Bacteroides_fragilis

Bacteroides_thetaiotaomicron

Binary_cloning_vector_pAC161

Binary_vector_pCS

Binary_vector_pCS4-BASK

Binary_vector_pSUN

Binary_vector_pSUNG

Bordetella_bronchiseptica

Chryseobacterium_indologenes

Citrobacter_freundii

Cloning_vector_pADIS1

Cloning_vector_pGABI1

Cloning_vector_pK-Sul1

Cloning_vector_pPSX

Corynebacterium_amycolatum

Corynebacterium_diphtheriae

Corynebacterium_glutamicum

Corynebacterium_resistens

Corynebacterium_sp._L2-79-05

Corynebacterium_striatum

Cronobacter_sakazakii

Delftia_sp._SL20

Desulfurispirillum_indicum

Edwardsiella_tarda

Enterobacter_aerogenes

Enterobacter_asburiae

Enterobacter_cloacae

Enterobacter_hormaechei

Enterobacter_ludwigii

Enterobacteriaceae_bacterium_SL1

Enterococcus_faecalis

Enterococcus_faecium

Escherichia_coli

Klebsiella_oxytoca

Klebsiella_pneumoniae

Klebsiella_sp._25

Klebsiella_sp._49

Klebsiella_sp._52

Kluyvera_ascorbata

Kluyvera_georgiana

Leclercia_adecarboxylata

Microbacterium_foliorum

Microbacterium_phyllosphaerae

Morganella_morganii

Mycobacterium_abscessus

Mycobacterium_africanum

Mycobacterium_bovis

Mycobacterium_canettii

Mycobacterium_fortuitum

Mycobacterium_tuberculosis

Ochrobactrum_sp._45C2

Plasmid_NR79

Plasmid_R388

Plasmid_R46

Plasmid_R974

Plasmid_pBP60-1

Plasmid_pBWH77

Plasmid_pLMO20

Proteus_mirabilis

Proteus_vulgaris

Providencia_rettgeri

Providencia_stuartii

Pseudomonas_aeruginosa

Pseudomonas_putida

Pseudomonas_sp._91S1

Pseudomonas_sp._Tik3

Raoultella_planticola

Raoultella_terrigena

Riemerella_anatipestifer

Salmonella_enterica

Serratia_liquefaciens

Serratia_marcescens

Shigella_dysenteriae

Shigella_flexneri

Shigella_sonnei

Shigella_sp._44

Shigella_sp._SHV-71

Sphingobacterium_sp._PM2-P1-29

Sphingomonas_sp._MM-1

Syntrophus_aciditrophicus

Thiomonas_intermedia

Thiomonas_sp._3As

Vibrio_cholerae

Vibrio_fluvialis

Yersinia_pestis

bacterium_36B

synthetic_construct

uncultured_Ochrobactrum_sp.

uncultured_Pseudomonas_sp.

uncultured_bacterium

uncultured_bacterium_HH1107

unidentified_cloning_vector
